# The correlation between the costs and clinical benefits of PD-1/PD-L1 inhibitors in malignant tumors: An evaluation based on ASCO and ESMO frameworks

**DOI:** 10.3389/fphar.2023.1114304

**Published:** 2023-02-23

**Authors:** Shen Lin, Yaping Huang, Liangliang Dong, Meiyue Li, Yahong Wang, Dian Gu, Wei Wu, Dongni Nian, Shaohong Luo, Xiaoting Huang, Xiongwei Xu, Xiuhua Weng

**Affiliations:** ^1^ Department of Pharmacy, The First Affiliated Hospital of Fujian Medical University, Fuzhou, China; ^2^ National Regional Medical Center, Department of Pharmacy, Binhai Campus of the First Affiliated Hospital, Fujian Medical University, Fuzhou, China; ^3^ Department of Pharmacy, Clinical Oncology School of Fujian Medical University, Fujian Cancer Hospital, Fuzhou, China; ^4^ Department of Pharmacy, Gansu Provincial Hospital, Lanzhou, China; ^5^ College of Pharmacy, Fujian Medical University, Fuzhou, China; ^6^ Institute for Health and Aging, University of California, San Francisco, San Francisco, CA, United States

**Keywords:** PD-1/PD-L1 inhibitors, malignant tumors, cost-benefit analysis, ASCO-VF framework, ESMO-MCBS framework

## Abstract

**Background:** Life expectancy for patients with malignant tumors has been significantly improved since the presence of the programmed cell death protein-1/programmed cell death protein ligand-1 (PD-1/PD-L1) inhibitors in 2014, but they impose heavy financial burdens for patients, the healthcare system and the nations. The objective of this study was to determine the survival benefits, toxicities, and monetary of programmed cell death protein-1/programmed cell death protein ligand-1 inhibitors and quantify their values.

**Methods:** Randomized controlled trials (RCTs) of PD-1/PD-L1 inhibitors for malignant tumors were identified and clinical benefits were quantified by American Society of Clinical Oncology Value Framework (ASCO-VF) and European Society for Medical Oncology Magnitude of Clinical Benefit Scale (ESMO-MCBS). The drug price in Micromedex REDBOOK was used to estimate monthly incremental drug costs (IDCs) and the correlation between clinical benefits and incremental drug costs of experimental and control groups in each randomized controlled trial, and the agreement between two frameworks were calculated.

**Results:** Up to December 2022, 52 randomized controlled trials were included in the quantitative synthesis. All the randomized controlled trials were evaluated by American society of clinical oncology value framework, and 26 (50%) met the American society of clinical oncology value framework “clinical meaningful value.” 49 of 52 randomized controlled trials were graded by European society for medical oncology magnitude of clinical benefit scale, and 30 (61.2%) randomized controlled trials achieved European Society for Medical Oncology criteria of meaningful value. *p*-values of Spearman correlation analyses between monthly incremental drug costs and American society of clinical oncology value framework/European society for medical oncology magnitude of clinical benefit scale scores were 0.9695 and 0.3013, respectively. In addition, agreement between two framework thresholds was fair (*κ* = 0.417, *p* = 0.00354).

**Conclusion:** This study suggests that there might be no correlation between the cost and clinical benefit of programmed cell death protein-1/programmed cell death protein ligand-1 inhibitors in malignancy, and the same results were observed in subgroups stratified by drug or indication. The results should be a wake-up call for oncologists, pharmaceutical enterprises and policymakers, and meanwhile advocate the refining of American Society of Clinical Oncology and European Society for Medical Oncology frameworks.

## Introduction

Survival benefits of patients with a malignant tumor have been improved significantly over the years, partially attributed to the employment of novel anti-cancer therapies. Recent success in immunotherapy propels cancer treatment to an exciting new era after traditional chemotherapy and targeted therapy (Chen et al., 2019). To date, approximately 4000 clinical trials focusing on programmed cell death protein-1/programmed cell death protein ligand-1 (PD-1/PD-L1) inhibitors have been carried out in at least 20 types of cancer, including both solid and hematological tumors; the total number of subjects worldwide is more than 20,000 (Chen et al., 2020). For the moment, approximately six PD-1/PD-L1 inhibitors are commonly used in clinical practice: Nivolumab, Pembrolizumab, Atezolizumab, Avelumab, Durvalumab, and Cemiplimab. These PD-1/PD-L1 inhibitors are demonstrated to have the preeminent potential for long-term survival, but along with dramatic high drug costs. Although the rapid development of novel therapies has provided insights into the future direction of treatments for malignancy, the high cost of cancer treatment has become a major concern for patients and the society. The financial toxicity may lead to psychosocial distress, poor quality of life (QOL), and worse patient outcomes. Thus, the focus that if the survival benefit and living quality are in proportion to the economics expenditure has been in the spotlight (Goulart, 2016).

However, it is always hard to objectively quantify therapy value and clinical benefit. It is commendable that the American Society of Clinical Oncology Value Framework (ASCO-VF) (Schnipper et al., 2015; Schnipper et al., 2016) and the European Society for Medical Oncology Magnitude of Clinical Benefit Scale (ESMO-MCBS) (Cherny et al., 2017a; Cherny et al., 2017b) have been proposed as evaluation frameworks to analyze survival, toxicity, and QOL of solid tumor patients. These two frameworks were both proposed successively in 2015 and refined in 2016 and 2017, respectively. Since the release of the first research refer to evaluate the clinical benefit and expenditure of solid tumor by using ASCO-VF and ESMO-MCBS frameworks in 2017, several similar studies were conducted in France, Canada, Switzerland Korea and so on (Del Paggio et al., 2017; Vivot et al., 2017; Saluja et al., 2018; Vokinger et al., 2020; Ha et al., 2022). The aforementioned studies aimed to evaluate the value of anti-cancer drugs and help patients and physicians to draw informed comparisons between different cancer treatments. Two tools are increasingly being used to assess the extent to which the magnitude of clinical benefit in these settings is associated with modern drug costs.

Thus, this study attempted to employ the ASCO-VF and the ESMO-MCBS to describe the clinical benefit of all approved PD-1/PD-L1 inhibitors for treating malignant tumors, and calculated the unit time cost of each agent, so as to explore a correlation between clinical benefit and price of drugs, likewise the correlation analyses in the subgroups of different agents or indications. Furthermore, consistency evaluation of two value frameworks was also computed.

## Materials and methods

### Identification of study cohort

PubMed was searched from the inception of a database to December 2022 to identify all the phase III randomized controlled trials (RCTs) in treatment with malignant tumors involving approved PD-1/PD-L1 inhibitors (Nivolumab, Pembrolizumab, Atezolizumab, Durvalumab, Avelumab and Cemiplimab), by the terms of drug names and clinical trials [i.e., nivolumab (Title/Abstract) AND clinical trial (Title/Abstract)]. Phase III RCTs registered on the clinicaltrials.gov website were also incorporated. Abstracts and methods of each trial were reviewed to identify the eligible cohort of trials according to inclusive criteria that RCTs could be analyzed with ASCO-VF or ESMO-MCBS, and the clinical benefit of the experimental groups should be preferred over the control groups. Analyses of patient-reported outcomes only assessing the QOL of the corresponding RCTs were also included. The following research were not taken into account: the secondary, subset, or systematic reviews; phase I, II or IV trials or animal studies; trials focused on other objectives including pharmacokinetics, drug dosing schedules, iconography, biomarkers, modeling, etc; non-trial-based papers like trial introduction; and trials that written by non-English articles. The study was conducted independently by two authors (YH and SL), and discrepancies were resolved by consensus in the presence of a third investigator (XW).

### ASCO-VF and ESMO-MCBS scoring

Gains in a survival endpoint and adjustments by toxicity and QOL in scores or grades were quantified by ASCO-VF (Version 2), or ESMO-MCBS (Version 1.1), or both if data allowed. The clinical benefit score of ASCO-VF is based on the point estimate of the hazard ratio (HR) of a couple of clinical endpoints covering overall survival (OS), progression-free survival (PFS), and response rates (RR), which is subtracted from 1 and the result is multiplied by 100 to derive the preliminary score. For toxicity assessment, both the number of the occurred case and the frequency (i.e., ≥10%, <10%, ≥5%, <5%) of all grades’ adverse events are correspondingly assigned “points”, which are applied to formulaically figure up the increment of the experimental group against the control group to derive an adjustment of the score (i.e., ±20 points maximum adjustment). For QOL, ASCO-VF allows an award of 10 points if a statistically significant improvement in QOL is reported but no deduction due to detrimental QOL. Besides, ASCO-VF includes bonus points for a “tail of the curve effect” (16–20 points), palliation of symptoms (10 points), and treatment-free intervals (a percentage-calculated improvement). The final ASCO-VF scores are the sum of above items (possible range −20–180) (Schnipper et al., 2016). ASCO-VF does not explicitly define “meaningful clinical benefit” scores, so the median score was used to determine meaningful clinical benefit according to the suggestion of reference (Del Paggio et al., 2017).

In the ESMO-MCBS grading system, the lower limits of the 95% CI of the HR of survival outcomes are used to determine a particular grade in a pre-specified manner, which is downgraded if pre-specified toxic effects are explicitly outlined in the experimental group with specifically, statistically significant incremental rates like “Toxic death >2%,” “Cardiovascular ischemia >2%,” “Grade 3 neurotoxicity >10%” and so on. For QOL assessment, upgrading or downgrading are allowed base on the improvement or deterioration of QOL. Ultimately, ESMO-MCBS grades are ranked from 1 to 5 for the advanced disease setting, and C, B, or A for the curative setting. ESMO-MCBS defines “meaningful clinical benefit” as a grade of 4, 5 or B, A (Cherny et al., 2017b).

### Incremental drug cost

To assess the monthly cost of therapeutic regimen including the cost of all anticancer drugs in the study regimen, we used the United States average wholesale prices (AWP) for drugs from the RedBook (IBM Micromedex, Armonk, NY, United States). Monthly costs were calculated over an average of 28 days based on the dosage schedule in all eligible trials for a patient weighing 70 kg with a body surface area of 1.86 m^2^ and creatinine clearance of 100 mL/min (Wan et al., 2019). Ultimately, incremental monthly drug costs between the experimental and control groups were reported. All therapeutic regimens were adjusted to provide the price per 4-week period.

### Statistical analysis

Study data like ASCO-VF and ESMO-MCBS scores, and incremental cost were mainly statistically described with median values, the 25th and the 75th percentile basing on treatment purposes, agents or indications. The scores of each trial were presented as a histogram. Spearman’s rank correlation coefficient (*r*) was calculated to assess the association between non-normally distributed data or ordinal data, such as costs and scores, which were showed by scatterplots or boxplots. Mann-Whitney *U* test was performed to describe the correlation between cost data and clinical benefit thresholds, shown as boxplots. Agreement between ASCO-VF and ESMO-MCBS in clinical benefits of RCTs was calculated *via* Cohen *κ* statistics, by which the result was between 0 and 1 (0 indicates agreement equivalent to chance and 1 indicates perfect agreement) (Cohen, 1960). No quantized analysis was made for ESMO-MCBS grades in the curative setting RCTs because its grades are non-numerical.

All statistical analyses were conducted in R (version 4.1.0) using ggplot2 (version 3.2.0) for plots. *p*-values of less than 0.05 were considered statistically significant.

## Results

### Overview and characteristics of RCTs

**FIGURE 1:**
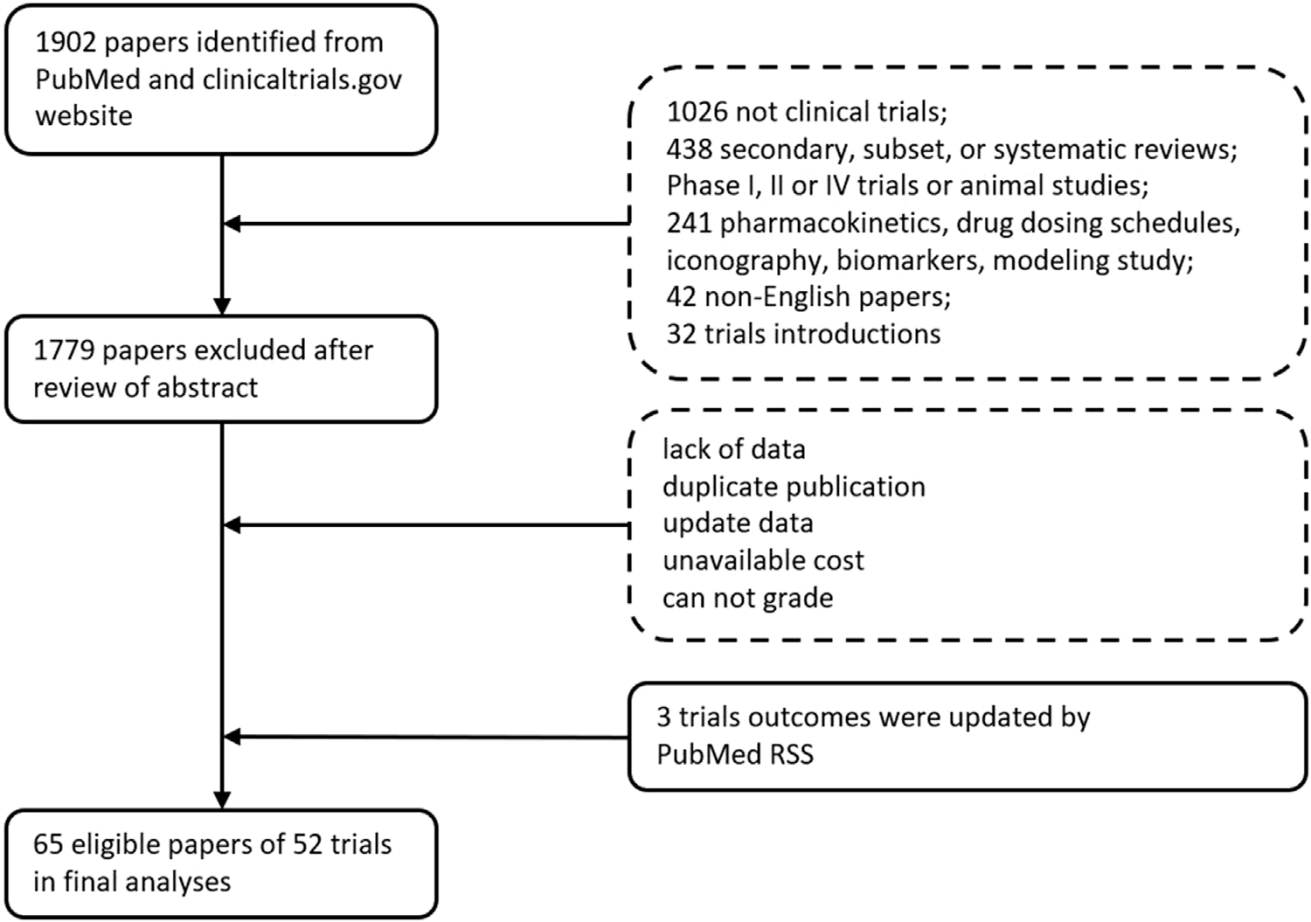
Identification of RCTs of all therapy in six immune checkpoint inhibitors.

A total of 101 RCTs were initially identified. After excluding trials that failing to meet inclusion criteria, 65 papers of 52 phase III RCTs were analyzed containing six PD-1/PD-L1 inhibitors: 17 RCTs for pembrolizumab, 14 RCTs for nivolumab, 14 RCTs for atezolizumab, 4 RCTs for durvalumab, 2 RCTs for avelumab, 1 RCTs for cemiplimab (Figure 1). The RCTs covered 12 indications, among which, 19 RCTs were used for non-small cell lung cancer (NSCLC), 7 for melanoma, 5 for breast cancer, 5 for renal cell cancer, 4 for urothelial cancer, 3 for gastric cancer, 2 for hepatocellular cancer, 2 for head-and-neck squamous cell carcinoma, 2 for small cell lung cancer (SCLC), 1 for colorectal cancer, 1 for glioblastoma, and 1 for malignant pleural mesothelioma. All of eligible papers were listed in the Supplementary Material.

### Frameworks scores

Of 52 RCTs, 4 were the curative setting, and the others were the advanced setting. All-inclusive RCTs were eligible for assessment by the ASCO-VF, and among which 49 RCTs were also eligible for ESMO-MCBS assessment.

ASCO-VF scores ranged from 0.40 to 86.71 (Supplementary Table S1). The scores were not normally distributed and therefore, were described in terms of medians and quartiles. The median ASCO-VF score of 52 RCTs was 39.81 (IQR 18.23–56.54), with 26 trials below and 26 trials above (Supplementary Figure S1). For the 48 palliative trials, 24 fell below the threshold and 24 were above the threshold (Median 40.16, IQR 21.16-56.79), whereas for the 4 curative trials, two felled below and the other two were above (Median 24.10, IQR 16.99-36.10).

For the assessment of ESMO-MCBS, among 49 RCTs, 19 trials felled below the “meaningful benefit” score, 30 were above (Supplementary Figure S1). For the 46 palliative trials, 18 fell below and 28 were above the threshold. In the 3 curative trials, 1 fell below and 2 were above the threshold. Median scores and quartiles of RCTs with different indications and agents were presented in Supplementary Table S1.

### Relation between cost and value of drug

**FIGURE 2:**
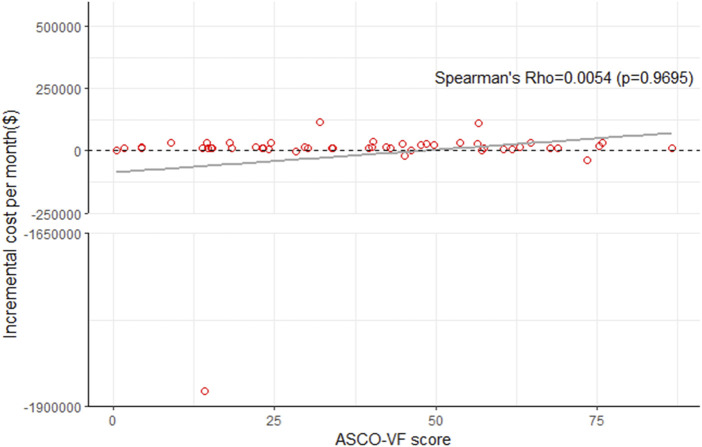
Scatterplot of six immune checkpoint inhibitors between ASCO-VF scores and incremental cost per month in all RCTs.

The incremental monthly drug costs (the cost of the experimental group minus the cost of the control group) of PD-1/PD-L1 inhibitors and the ASCO-VF score were not statistically significant correlated in all trials (Spearman’s *ρ* = 0.0054, *p* = 0.9695, Figure 2), the subgroup of palliative treatments (*ρ* = −0.0396, *p* = 0.3946), and curative treatments (*ρ* = 0.6324, *p* = 0.184) (Supplementary Figure S2).

**FIGURE 3:**
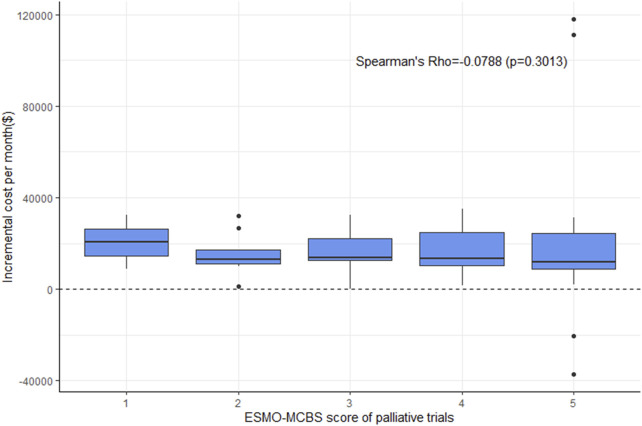
Boxplot of correlation between ESMO-MCBS scores and incremental cost per month in palliative trials.

**FIGURE 4:**
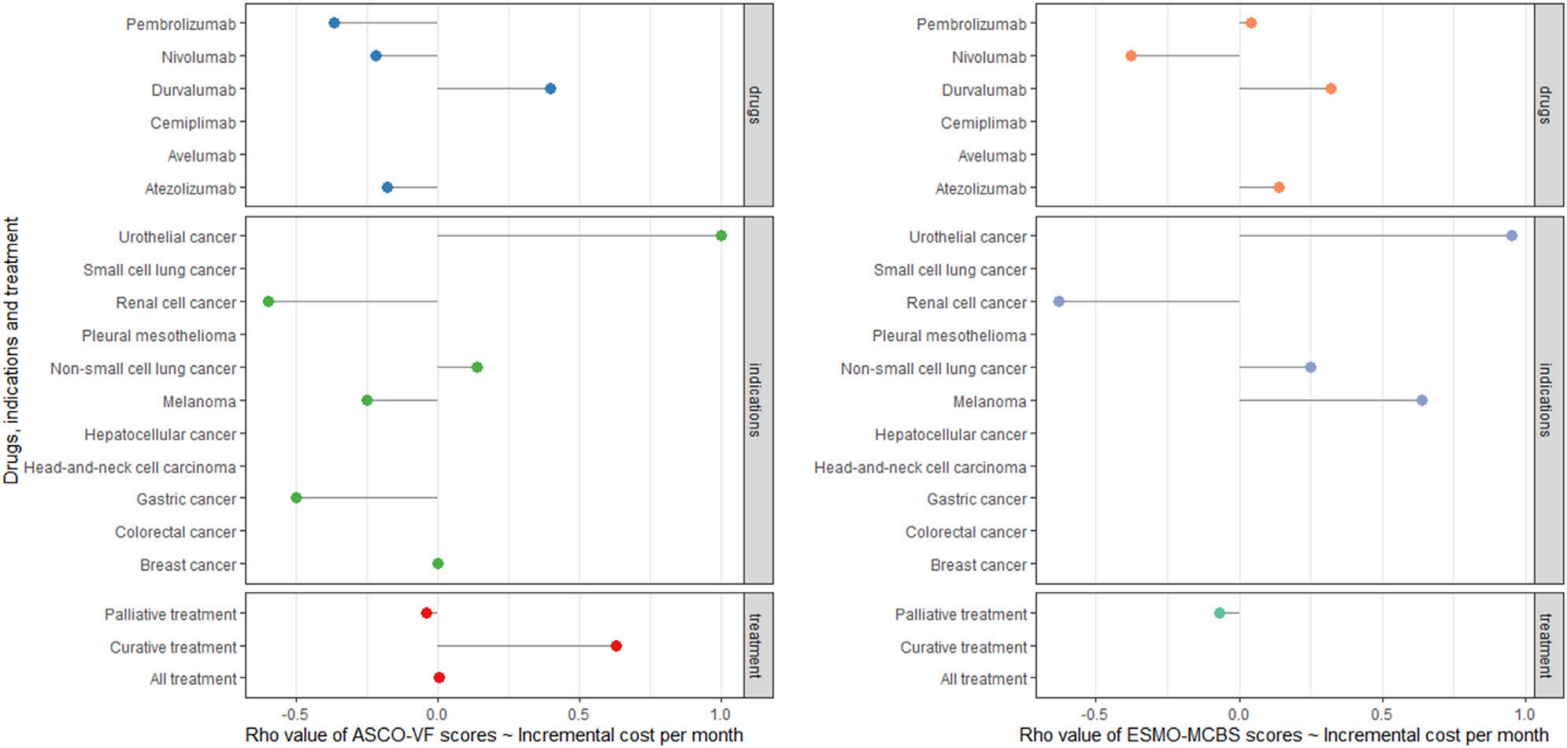
Cleverland of correlation analyses among ASCO-VF scores, ESMO-MCBS scores and incremental cost in six PD-1/PD-L1 inhibitors and 11 indications.

For ESMO-MCBS grades, no statistically significant association was also noted in the palliative setting (*ρ* = −0.0788, *p* = 0.3013) (Figure 3). Stratified by indications or drugs, no statistically correlations were found between either framework and costs (*p >* 0.05) (Figure 4). Correlation analysis could not be conducted in curative setting due to its non-consecutive numerical data of grades C, B and A.

The incremental monthly drug costs of trials that did not meet the ASCO-VF threshold for meaningful benefit was slightly lower than that of met the meaningful benefit [$12504 (IQR 11902 to 15451) vs. $13392 (IQR 9391 to 26681); *p* = 0.8444], while the opposite result was observed when used ESMO-MCBS framework [not met the threshold $12948 (IQR 10435 to 23517)vs. met the threshold $12499 (IQR 9629 to 24875); *p* = 0.9014] (Supplementary Figure S3). Neither result was statistical significance.

### Agreement of frameworks

When comparing the RCTs scores using the framework-specified thresholds, Cohen *κ* statistic was calculated as 0.417 (*p* = 0.00354), which suggested a moderate agreement between ASCO-VF and ESMO-MCBS thresholds. In subgroup analyses, within the palliative subset, the *κ* score (0.421, *p* = 0.00426) was similar to that in the total cohort, whereas in curative setting of trials, the *κ* score was weaker than the total cohort (0.333, *p* = 0.564).

## Discussion

Cancer drug innovation has been accelerating since entering the 21st century. The number of novel cancer drugs approved in 2005–2015 was over 8 times more than that approved in 1975–1985 (66 vs. 8), and the average annual growth rate of total cancer drug expenditure was 7.6%, 3.6 times more than the average annual growth rate of nominal United States Gross Domestic Product (Lichtenberg, 2020). In the context of limited medical resources, it is essential to evaluate the correlation between clinical benefit and medical expenditure. To the best of our knowledge, this was the first study that applied ASCO-VF and ESMO-MCBS to assess the clinical benefit of all approved PD-1/PD-L1 inhibitors comprehensively. Both frameworks demonstrated that only nearly half of the eligible trials (26 of 52 trials in ASCO-VF and 30 of 49 in ESMO-MCBS) had met the “meaningful clinical benefit” thresholds correspondingly, which suggested that quite a lot of RCTs only demonstrated subtle clinical benefits. Furthermore, there was no statistically significant correlation between drug price and the clinical benefit in all trials, even in the subgroups of different indications/different agents, which revealed that high prices might not definitely yield the equivalent benefit.

Previously, two prior studies showed no significant association between clinical benefit and the price of new FDA-approved anti-cancer drugs with initial indications in the United States from 2000 to 2017, using both ASCO-VF and ESMO-MCBS (Vivot et al., 2017; Vokinger et al., 2020). The result of our study, which focused on PD-1/PD-L1 inhibitors, were consistent with two prior studies and partial presented the weak association between clinical benefit and the drug price in all anti-cancer drugs. One prominent reason attributed to this situation might be that these novel agents are always highly priced by pharmaceutical enterprises within patent protection. As per the Tufts Center for the Study of Drug Development in 1975, pharmaceutical industries expended 100 million dollars for the research and development of the FDA-approved drug, which had surged to $1.3 billion in 2005 stupendously (Kunnumakkara et al., 2019). In order to repay their high and risky investment cost, pharmaceutical companies would charge more for their products, which may be the partial cause for the high sale prices of drugs outweigh their clinical efficacy. Besides, the inaccurate evaluation of drug efficiency is another contributor. Many drugs get approval from the FDA in an expedited regulatory pathway (called accelerated approval) on the basis of existing trial endpoints at that time, which probably exaggerates the clinical benefit and safety of these drugs provisionally. Some drugs or indications were withdrawn from the market after reevaluation in post-marketing studies (Wilson et al., 2013), such as the indications that pembrolizumab in second-line treatment of SCLC, nivolumab in second-line treatment of SCLC, nivolumab in second-line treatment of BRAF-positive melanoma, atezolizumab in urothelial cancer and so forth. A report published by FDA indicated that from 11 December 1992 to 31 May 2017, 5% of 93 indications of oncology were withdrawn in light of post-approval trials results (Beaver et al., 2018), which suggests the accuracy of evaluating anti-cancer deserves more attention. In a word, the high drug cost and the uncertainty of clinical benefit work together to the no association between them.

Two value frameworks were applied in this study, and a moderate agreement was found between them. There is a controversy exist in the agreement of these two frameworks (Cheng et al., 2017; Del Paggio et al., 2017; Vivot et al., 2017; Cherny et al., 2019; Jiang et al., 2020), which are not surprising given the differences in their construction and scoring criteria. First, major factors contributing to discrepancy are different methods of evaluating relative and absolute gain for OS and PFS, applying toxicity penalties, and crediting the tail of the curve gains. By these methods, the ASCO-VF tends to generate lower clinical benefit scores in comparison to ESMO-MCBS. Second, the frameworks differ in their criteria for awarding bonus credits for long-term survival gain. The ASCO-VF criteria awards bonus points on the basis of a 50% or greater improvement at the time point that is twice the comparator median survival time on the survival curves. ESMO-MCBS credits an adjustment grade if there is a long-term plateau in specified time points of the survival curves. Third, both frameworks award bonus scores for treatments that reduce toxicity, but their approaches differ, which have been described in methods. Distinctness of awarding bonus in ASCO-VF and ESMO-MCBS generated the gap in clinical benefit scores as well. Although the tools are imperfect, they have been at the forefront of evaluating the relation between clinical benefit and cost for many years.

From the perspective of society, growing expenditures on anticancer drugs can potentially occupy the investment of other life–saving medicine, and contribute to the unbalanced allocation of medical resources. Virtually, many drugs like anti-cardiovascular diseases drugs are available as generics or “me-too” that are defined a new pharmaceutical compound with a known pharmaceutical class of treatment, and increasing competition consequently led to diminishing overall costs in these pharmaceutical companies, while most the anticancer agents are the first-in-class agents. During 1970–2000, the life expectancy of Americans increased on average by 6 years; only 6 months were attributed to antineoplastic therapies, while over 4 years were attributed to cardiovascular disease (Lenfant, 2003). A horrendous disequilibrium between prices and survival benefits causes a dire socioeconomics cost and puts a substantial burden on the medication budgets of public health organizations. Therefore, it has profound meaning to assess the survival benefits and economy investment to re-allocate medical resources.

This study has several limitations. Firstly, we only evaluated available trials published to assess the ASCO-VF and the ESMO-MCBS scores to date. Within a trial, outcomes of long-term follow-up and the further pooled estimate of efficacy result would evolve with time, which lead to the dynamics of clinical benefit scores of drugs (Schnipper et al., 2016). Similarly, due to the data availability, agents that have not been approved or whose wholesale prices are not accessible were not included in our study. We also excluded studies written in non-English languages. All these incomplete and inconclusive data would give rise to biases in subsequent analysis. Secondly, different from ESMO-MCBS, ASCO-VF does not provide its own “meaningful clinical benefit” threshold, so we use the median value of ASCO-VF scores for comparison according to the reference, which may partly contribute to the moderate agreement between ASCO-VF and ESMO-MCBS. In addition, in this study, only monthly incremental drug costs were considered, but treatment duration might affect the total cost differences between the experimental group and the control group, whichever probably have predefined courses. However, most of the included trials were palliative treatment, and the calculated incremental costs likely represent approximately 90% of the total treatment course increment cost, so monthly incremental drug costs were a close approximation reflection so long as response to treatment continues (Mittmann et al., 2009; Bradbury et al., 2010; Del Paggio et al., 2017). Thirdly, due to the limitation of sample size and research design, many phase III clinical trials in malignancy have relatively wide 95% CI. Based on the instructions of these two frameworks, point estimation of HRs was utilized in ASCO-VF framework tool, which would add uncertainty to the scores. The ASCO-VF should be planned revised and dynamically updated upon recognition of expanding needs and shortcomings identified. While in the latest version of ESMO-MCBS framework (Version 1.1), the lower limit of 95% CI is adopted for a required HR, and the absolute survival gain is taken into account, potentially balancing this uncertainty. Finally, understanding degree of frameworks among different investigators would be reflected in the research. Although this analysis was performed by three investigators, some trivial discrepancy could not be averted. A modified framework or updated trial results are expected to assist in evaluating the cost-benefit of drugs accurately, and shared decision making regarding the options available to oncologists and patients.

## Conclusion

This research indicated that on account of ASCO-VF and ESMO-MCBS frameworks, no correlation between the costs and clinical benefits of PD-1/PD-L1 inhibitors was present in treating malignant tumors, and the same results were observed in subgroups stratified by drugs or indications. In addition, the agreement between two framework thresholds was moderate. The result suggests that a comprehensive cost-benefit assessment of novel cancer drugs should guide oncological drug approval in public healthcare organizations, and methods to control and limit drug cost should be coordinated among healthcare providers, pharmaceutical companies, and policymakers. Meanwhile, the refining of ASCO and ESMO frameworks might be addressed to facilitate the standard assessment of clinical benefit of anti-cancer drugs.

## Data Availability

The original contributions presented in the study are included in the article/Supplementary Material, further inquiries can be directed to the corresponding authors.
