# Supplementary material for: The correlation between the costs and clinical benefits of PD-1/PD-L1 inhibitors in malignant tumors: An evaluation based on ASCO and ESMO frameworks
Source: Front Pharmacol. 2023 Feb 23;14:1114304. doi: 10.3389/fphar.2023.1114304 (PMC9995671; doi:10.3389/fphar.2023.1114304)
Supplement: Supplementary file 1 [file DataSheet1.docx]

**Supplementary Materials**

**Figure S1.** Distribution of ASCO-VF scores and ESMO-MCBS benefit threshold in a cost-benefit analysis cohort of RCTs of six immune checkpoint inhibitors.

**Figure S2.** Scatterplot of correlation between ASCO-VF scores and incremental cost per month in palliative or curative trails of six immune checkpoint inhibitors.

**Figure S3.** Boxplots of association between ASCO-VF or ESMO-MCBS scores and incremental cost per month of six immune checkpoint inhibitors.

**Table S1.** Description of ASCO-VF, ESMO-MCBS scores and incremental cost of RCTs in six PD-1/PD-L1 inhibitors and 11 indications

| **Stratify (No. of RCTs)** | **ASCO-VF scores** | | | **ESMO-MCBS scores** | | | **Incremental cost per month ($)** | | |
| --- | --- | --- | --- | --- | --- | --- | --- | --- | --- |
|  | **Median (IQR)** | **Min** | **Max** | **Median** | **Min** | **Max** | **Median (IQR)** | **Min** | **Max** |
| **All treatment** (52) | 39.81 (18.23–56.54) | 0.40 | 86.71 | - | - | - | 12,817.09 (9,997.74-26,837.97) | -1,878,303.73 | 117,965.99 |
| Curative treatment (4) | 24.10 (16.99-36.10) | 14.05 | 53.73 | - | - | - | 22,672.63 (-459,728.65-32,215.55) | -1,878,303.73 | 32,215.55 |
| Palliative treatment (48) | 40.16 (21.16-56.79) | 0.40 | 86.71 | 4.0 | 1.0 | 5.0 | 12,504.48 (10,041.30-26,681.65) | -37,139.23 | 117,965.99 |
| **Drugs** | | | | | | | | | |
| Avelumab (2) (1,2) | 25.87 (3.99-27.75) | 22.11 | 29.63 | 3.5 | 3.0 | 4.0 | 15,451.99 (14,736.49-16,167.48) | 14,021.00 | 16,882.97 |
| Atezolizumab (14) (3–16) | 18.31 (14.68-33.84) | 0.40 | 61.88 | 3.0 | 2.0 | 5.0 | 12,504.48 (10,271.61-12,504.48) | 1,316.88 | 13,129.70 |
| Durvalumab (4) (17–21) | 39.81 (30.08-46.99) | 1.70 | 67.74 | 3.5 | 1.0 | 5.0 | 11183.12 (9,604.72-12,722.40) | 8,817.72 | 13,392.00 |
| Nivolumab (14) (22–43) | 46.27 (42.30-63.00) | 4.35 | 86.71 | 5.0 | 2.0 | 5.0 | 8586.89 (312.45-12,104.96) | -1,878,303.73 | 14,026.24 |
| Pembrolizumab (17) (44–64) | 47.68 (28.23-56.59) | 8.88 | 75.92 | 4.0 | 1.0 | 5.0 | 32215.55 (27,306.91-32,215.55) | 21,211.92 | 117,965.99 |
| Cemiplimab (1) (65) | 57.49 (57.49-57.49) | 57.49 | 57.49 | 4.0 | 4.0 | 4.0 | 10461.32 (-) | - | - |
| **Indication** | | | | | | | | | |
| Breast cancer (5) | 15.14 (14.40-17.97) | 13.84 | 30.22 | - | - | - | 13129.70 (13,129.70-17,901.16) | 13,129.70 | 32,215.55 |
| Melanoma (7) | 18.31 (11.47-47.50) | 4.35 | 73.48 | - | - | - | 13800.80 ( -13,548.96-23,015.24) | -1,878,303.73 | 117,965.99 |
| Gastric cancer (3) | 42.30 (30.15-45.45) | 18.00 | 48.59 | 2.0 | 2.0 | 2.0 | 26681.65 (20,353.94-29,331.24) | 14,026.24 | 31,980.82 |
| Urothelial cancer (4) | 22.47 (12.57-38.41) | 4.40 | 64.76 | 2.5 | 2.0 | 5.0 | 13262.74 (12,282.94-1,8348.37) | 11,618.30 | 31,330.46 |
| Renal cell cancer (5) | 40.24 (24.00-45.18) | 22.11 | 57.12 | 4.0 | 3.0 | 5.0 | 7352.85 (108.41-16,882.97) | -20,643.25 | 35,077.87 |
| Hepatocellular cancer (2) | 68.90 (65.39-72.41) | 61.88 | 75.92 | 4.0 | 4.0 | 4.0 | 18977.61 (12,358.65-25,596.58) | 5,739.68 | 32,215.55 |
| Small cell lung cancer (2) | 31.62 (27.39-35.85) | 23.16 | 40.08 | 2.5 | 2.0 | 3.0 | 12948.24 (12,726.36-13,170.12) | 12,504.48 | 13,392.00 |
| Non-small cell lung cancer (19) | 43.00 (29.15-57.08) | 0.40 | 86.71 | 4.0 | 1.0 | 5.0 | 12187.14 (10,164.18-16,858.20) | 1,316.88 | 111,212.45 |
| Head-and-neck cell carcinoma (2) | 52.67 (48.75-56.58) | 44.83 | 60.50 | 4.0 | 3.0 | 5.0 | 18737.79 (13,260.03-24,215.54) | 7,782.28 | 29,693.30 |
| Colorectal cancer (1) | - | - | - | - | - | - | 22443.40 (-) | - | - |
| Pleural mesothelioma (1) | - | - | - | - | - | - | -71.61 (-) | - | - |

Table S2. Characteristic of Trials and drug prices

| Trial | Tumor site | Experimental  therapy | Control therapy | Clinical Endpoint | Incremental cost ($US) * |
| --- | --- | --- | --- | --- | --- |
| IMpassion130 | Breast cancer | Atezolizumab+chemotherapy q4w | Placebo+chemotherapy q4w | OS | 13129.70 |
| IMspire150 | Melanoma | Atezolizumab+vemurafenib+ cobimetinib q3w | Placebo+vemurafenib+cobimetinib q3w | PFS | 10041.30 |
| IMbrave150 | HCC | Atezolizumab+bevacizumab q3w | Sorafenib bid | OS | 5739.68 |
| IMpower110 | NSCLC | Atezolizumab+chemotherapy q3w | Chemotherapy q3w | OS | 12504.48 |
| IMpower130 | NSCLC | Atezolizumab+chemotherapy q3w | Placebo+chemotherapy q3w | OS | 12504.48 |
| IMpower133 | SCLC | Atezolizumab+chemotherapy q3w | Placebo+chemotherapy q3w | OS | 12504.48 |
| IMpassion130 | Breast cancer | Atezolizumab+chemotherapy q4w | Placebo + chemotherapy q4w | OS | 13129.70 |
| IMpower150 | NSCLC | Atezolizumab+bevacizumab + chemotherapy q3w | Bevacizumab+chemotherapy q3w | OS | 12504.48 |
| NCT02008227 | NSCLC | Atezolizumab q3w | Docetaxel q3w | OS | 10962.52 |
| IMmotion151 | Renal cell cancer | Atezolizumab+bevacizumab q3w | Sunitinib qd q4/6w | OS | 7352.85 |
| IMpower132 | NSCLC | Atezolizumab q3w | Pemetrexed + cisplatin/carboplatin q3w | OS | 1316.8794 |
| IMvigor211 | Urothelial cancer | Atezolizumab q3w | Vinflunine/paclitaxel/docetaxel q3w | OS | 11618.30 |
| IMvigor130 | Urothelial cancer | Atezolizumab+chemotherapy q3w | Placebo+chemotherapy q3w | OS | 12504.48 |
| IMpassion031 | Breast cancer | Atezolizumab+chemotherapy q4w | Placebo+chemotherapy q4w | OS | 13129.70 |
| NCT02603432 | Urothelial cancer | Avelumab q2w+BSC | BSC | OS | 14021.00 |
| NCT02684006 | Renal cell cancer | Avelumab q2w+axitinib 5mg bid | Sunitinib qd q4/6w | PFS | 16882.97 |
| ARCTIC | NSCLC | Durvalumab q2w | Erlotinib qd  Gemcitabine q4w  Vinorelbine q4w | OS | 9867.05 |
| NCT02453282 | NSCLC | Durvalumab q4w | Paclitaxel+carboplatin q3w  Gemcitabine+cisplatin q3w  Gemcitabine+carboplatin q3w  Pemetrexed+cisplatin q3w  Pemetrexed+carboplatin q3w | OS | 8817.72 |
| CASPIAN | SCLC | Durvalumab+chemotherapy q3w*4, then q4w | Chemotherapy q3w | OS | 13392.00 |
| PACIFIC | NSCLC | Durvalumab q2w | Placebo q3w | OS | 12499.20 |
| KEYNOTE-407 | NSCLC | Pembrolizumab+chemotherapy q3w | Chemotherapy q3w | OS | 32215.55 |
| KEYNOTE-054 | melanoma | Pembrolizumab q3w | Placebo q3w | OS | 32215.55 |
| KEYNOTE-062 | Gastric Cancer | Pembrolizumab q3w | Cisplatin+fluorouracil q3w  Cisplatin+capecitabine bid d1-14 q3w | OS | 26681.65 |
| KEYNOTE-024 | NSCLC | Pembrolizumab q3w | Carboplatin+pemetrexed q3w  Cisplatin+pemetrexed q3w  Carboplatin+gemcitabine q3w  Cisplatin+gemcitabine q3w  Carboplatin+paclitaxel q3w | OS | 27306.91 |
| KEYNOTE-045 | urothelial cancer | Pembrolizumab q3w | Paclitaxel q3w  Docetaxel q3w  Vinflunine q3w | OS | 31330.46 |
| - | recurrent glioblastoma | Pembrolizumab q3w neoadjuvant and adjuvant therapy | Pembrolizumab q3w adjuvant therapy | OS | 32215.54 |
| KEYNOTE-006 | melanoma | Pembrolizumab q2w | Ipilimumab q3w | OS | 117965.99 |
| KEYNOTE-189 | NSCLC | Pembrolizumab q3w | Cisplatin/carboplatin+pemetrexed q3w, then pemetrexed q3w | OS | 21211.92 |
| KEYNOTE-240 | HCC | Pembrolizumab q3w | placebo q3w | OS | 32215.54 |
| KEYNOTE-522 | breast cancer | Pembrolizumab+chemotherapy q3w | Placebo+chemotherapy q3w | PFS | 32215.54 |
| KEYNOTE-177 | Colorectal Cancer | Pembrolizumab q3w | 5-fluorouracil–based therapy with or without bevacizumab or  cetuximab | PFS | 22443.40 |
| KEYNOTE-426 | renal cell cancer | Pembrolizumab q3w+axitinib bid | Sunitinib qd 4/2 q6w | OS | 35077.87 |
| KEYNOTE-355 | breast cancer | Pembrolizumab+chemotherapy q3w | Placebo+chemotherapy q3w | PFS | 32215.55 |
| KEYNOTE-042 | NSCLC | Pembrolizumab q3w | Carboplatin+paclitaxel/pemetrexed q3w | OS | 21971.58 |
| KEYNOTE-010 | NSCLC | Pembrolizumab q3w | Docetaxel q3w | OS | 111212.45 |
| KEYNOTE-040 | head-and-neck squamous cell carcinoma | Pembrolizumab q3w | Methotrexate qw+docetaxel q3w+cetuximab qw | OS | 29693.30 |
| KEYNOTE-061 | Gastric Cancer | Pembrolizumab q3w | Paclitaxel q4w | OS | 31980.82 |
| CHECKMATE-037 | melanoma | Nivolumab q2w | Dacarbazine q3w  Carboplatin+paclitaxel q3w | OS | 13800.80 |
| CHECKMATE-066 | melanoma | Nivolumab q2w | Dacarbazine q3w | OS | 13814.94 |
| CHECKMATE-067 | melanoma | Nivolumab q2w | Ipilimumab q3w | OS | -37139.22 |
| CHECKMATE-238 | melanoma | Nivolumab q2w | Ipilimumab q3w | RFS | -1878303.7280 |
| CHECKMATE-227 | NSCLC | Nivolumab q2w+ipilimumab q6w | Platinum-doublet Chemotherapy q3w | OS | 9391.50 |
| CHECKMATE-9LA | NSCLC | Nivolumab q2w+ipilimumab q3w+2 cycle chemotherapy q3w | Chemotherapy q3w | OS | 1464.6348 |
| CHECKMATE-017 | NSCLC | Nivolumab q2w | Docetaxel q3w | OS | 11858.39 |
| CHECKMATE-057 | NSCLC | Nivolumab q2w | Docetaxel q3w | OS | 11858.39 |
| CHECKMATE-078 | NSCLC | Nivolumab q2w | Docetaxel q3w | OS | 12187.14 |
| CHECKMATE-743 | malignant pleural mesothelioma | Nivolumab q2w+Ipilimumab q6w | Carboplatin+paclitaxel/pemetrexed q3w | OS | -71.6098 |
| CHECKMATE-025 | renal cell cancer | Nivolumab q2w | Everolimus qd | OS | -20643.25 |
| CHECKMATE-214 | renal cell cancer | Nivolumab+ipilimumab q3w, then nivolumab q2w | Sunitinib qd q4/6w | OS | 2108.41 |
| CHECKMATE-141 | head-and-neck squamous cell carcinoma | Nivolumab q2w | methotrexate qw+docetaxel q3w+cetuximab qw | OS | 7782.28 |
| ATTRACTION‑2 | Gastric Cancer | Nivolumab q2w | Placebo q2w | OS | 14026.23 |
| EMPOWER-Lung 1 | NSCLC | Cemiplimab q3w | Carboplatin+pemetrexed q3w  Cisplatin+pemetrexed q3w  Carboplatin+gemcitabine q3w  Cisplatin+gemcitabine q3w  Carboplatin+paclitaxel q3w Cisplatin+paclitaxel q3w | OS | 10461.32 |
| Drug | Strength | Unit price | Drug | Strength | Unit price |
| Atezolizumab | 60mg/1ml | 468.918(60mg) | Avelumab | 20mg/1ml | 200.304 (20mg) |
| Axitinib | 5mg | 332.56 (5mg) | Bevacizumab | 25mg/1ml | 189.46 (25mg) |
| Capecitabine | 500mg | 35.71 (500mg) | Cisplatin | 1mg/1ml | 1.30 (1mg) |
| Carboplatin | 10mg/1ml | 0.71 (10mg) | Cemiplimab | 50mg/1ml | 1583.4 (50mg) |
| Cetuximab | 2mg/1ml | 16.31 (2mg) | Dacarbazine | 200mg | 19.69 (200mg) |
| Docetaxel | 160mg/8ml | 22.95 (20mg) | Docetaxel | 20mg/1ml | 26.40 (20mg) |
| Durvalumab | 50mg/1ml | 446.398 (50mg) | Erlotinib | 150mg | 241 (150mg) |
| Everolimus | 5mg | 605.56 (5mg) | Fluorouracil | 50mg/1ml | 30.79 (50mg) |
| Gemcitabine | 100mg/1ml | 11.55 (100mg) | Ipilimumab | 5mg/1ml | 913.67 (5mg) |
| Methotrexate | 25mg/1ml | 7.55 (25mg) | Paclitaxel | 6mg/1ml | 0.537 (6mg) |
| Pemetrexed | 100mg | 905.48 (100mg) | Pembrolizumab | 25mg/1ml | 1510.11 (25mg) |
| Sorafenib | 200mg | 155.13 (200mg) | Sunitinib | 50mg | 844.36 (50mg) |
| Vemurafenib | 240mg | 44.12 (240mg) |  |  |  |

*Defined as (monthly cost of experimental regimen) – (monthly cost of control regimen). Placebo and BSC were considered to be $0. Costs were calculated assuming that patients weighed 70 kg or had a body surface area of 1·86 m² and creatinine clearance of 100 mL/minute. The median prices of the drug who have several generics and branded products were recorded. The average costs were calculated if there were several options of therapeutic regimen in the control group. All therapeutic regimens were adjusted to provide the price per 4-week period. Prices were obtained from RED BOOK.

**References**

1. Powles T, Park SH, Voog E, Caserta C, Valderrama BP, Gurney H, et al. Avelumab Maintenance Therapy for Advanced or Metastatic Urothelial Carcinoma. N Engl J Med. 2020 Sep 24;383(13):1218–30.

2. Motzer RJ, Penkov K, Haanen J, Rini B, Albiges L, Campbell MT, et al. Avelumab plus Axitinib versus Sunitinib for Advanced Renal-Cell Carcinoma. N Engl J Med. 2019 Mar 21;380(12):1103–15.

3. Schmid P, Rugo HS, Adams S, Schneeweiss A, Barrios CH, Iwata H, et al. Atezolizumab plus nab-paclitaxel as first-line treatment for unresectable, locally advanced or metastatic triple-negative breast cancer (IMpassion130): updated efficacy results from a randomised, double-blind, placebo-controlled, phase 3 trial. Lancet Oncol. 2020 Jan;21(1):44–59.

4. Gutzmer R, Stroyakovskiy D, Gogas H, Robert C, Lewis K, Protsenko S, et al. Atezolizumab, vemurafenib, and cobimetinib as first-line treatment for unresectable advanced BRAF(V600) mutation-positive melanoma (IMspire150): primary analysis of the randomised, double-blind, placebo-controlled, phase 3 trial. Lancet. 2020 Jun 13;395(10240):1835–44.

5. Finn RS, Qin S, Ikeda M, Galle PR, Ducreux M, Kim TY, et al. Atezolizumab plus Bevacizumab in Unresectable Hepatocellular Carcinoma. N Engl J Med. 2020 May 14;382(20):1894–905.

6. Herbst RS, Giaccone G, de Marinis F, Reinmuth N, Vergnenegre A, Barrios CH, et al. Atezolizumab for First-Line Treatment of PD-L1–Selected Patients with NSCLC. N Engl J Med. 2020 Oct 1;383(14):1328–39.

7. West H, McCleod M, Hussein M, Morabito A, Rittmeyer A, Conter HJ, et al. Atezolizumab in combination with carboplatin plus nab-paclitaxel chemotherapy compared with chemotherapy alone as first-line treatment for metastatic non-squamous non-small-cell lung cancer (IMpower130): a multicentre, randomised, open-label, phase 3 trial. Lancet Oncol. 2019 Jul;20(7):924–37.

8. Horn L, Mansfield AS, Szczęsna A, Havel L, Krzakowski M, Hochmair MJ, et al. First-Line Atezolizumab plus Chemotherapy in Extensive-Stage Small-Cell Lung Cancer. N Engl J Med. 2018 Dec 6;379(23):2220–9.

9. Schmid P, Adams S, Rugo HS, Schneeweiss A, Barrios CH, Iwata H, et al. Atezolizumab and Nab-Paclitaxel in Advanced Triple-Negative Breast Cancer. N Engl J Med. 2018 Nov 29;379(22):2108–21.

10. Socinski MA, Jotte RM, Cappuzzo F, Orlandi F, Stroyakovskiy D, Nogami N, et al. Atezolizumab for First-Line Treatment of Metastatic Nonsquamous NSCLC. N Engl J Med. 2018 Jun 14;378(24):2288–301.

11. Rittmeyer A, Barlesi F, Waterkamp D, Park K, Ciardiello F, von Pawel J, et al. Atezolizumab versus docetaxel in patients with previously treated non-small-cell lung cancer (OAK): a phase 3, open-label, multicentre randomised controlled trial. Lancet. 2017 21;389(10066):255–65.

12. Nishio M, Barlesi F, West H, Ball S, Bordoni R, Cobo M, et al. Atezolizumab Plus Chemotherapy for First-Line Treatment of Non-Squamous Non-Small Cell Lung Cancer: Results From the Randomized Phase III IMpower132 Trial. J Thorac Oncol. 2020 Dec 14;

13. Powles T, Durán I, van der Heijden MS, Loriot Y, Vogelzang NJ, De Giorgi U, et al. 11111Atezolizumab versus chemotherapy in patients with platinum-treated locally advanced or metastatic urothelial carcinoma (IMvigor211): a multicentre, open-label, phase 3 randomised controlled trial. Lancet. 2018 Feb 24;391(10122):748–57.

14. Galsky MD, Arija JÁA, Bamias A, Davis ID, De Santis M, Kikuchi E, et al. 11111Atezolizumab with or without chemotherapy in metastatic urothelial cancer (IMvigor130): a multicentre, randomised, placebo-controlled phase 3 trial. Lancet. 2020 May 16;395(10236):1547–57.

15. Mittendorf EA, Zhang H, Barrios CH, Saji S, Jung KH, Hegg R, et al. Neoadjuvant atezolizumab in combination with sequential nab-paclitaxel and anthracycline-based chemotherapy versus placebo and chemotherapy in patients with early-stage triple-negative breast cancer (IMpassion031): a randomised, double-blind, phase 3 trial. Lancet. 2020 Oct 10;396(10257):1090–100.

16. Motzer RJ, Powles T, Atkins MB, Escudier B, McDermott DF, Alekseev BY, et al. Final Overall Survival and Molecular Analysis in IMmotion151, a Phase 3 Trial Comparing Atezolizumab Plus Bevacizumab vs Sunitinib in Patients With Previously Untreated Metastatic Renal Cell Carcinoma. JAMA Oncol. 2022 Feb 1;8(2):275–80.

17. Planchard D, Reinmuth N, Orlov S, Fischer JR, Sugawara S, Mandziuk S, et al. ARCTIC: durvalumab with or without tremelimumab as third-line or later treatment of metastatic non-small-cell lung cancer. Ann Oncol. 2020 May;31(5):609–18.

18. Rizvi NA, Cho BC, Reinmuth N, Lee KH, Luft A, Ahn MJ, et al. Durvalumab With or Without Tremelimumab vs Standard Chemotherapy in First-line Treatment of Metastatic Non-Small Cell Lung Cancer: The MYSTIC Phase 3 Randomized Clinical Trial. JAMA Oncol. 2020 May 1;6(5):661–74.

19. Goldman JW, Dvorkin M, Chen Y, Reinmuth N, Hotta K, Trukhin D, et al. Durvalumab, with or without tremelimumab, plus platinum-etoposide versus platinum-etoposide alone in first-line treatment of extensive-stage small-cell lung cancer (CASPIAN): updated results from a randomised, controlled, open-label, phase 3 trial. Lancet Oncol. 2021 Jan;22(1):51–65.

20. Antonia SJ, Villegas A, Daniel D, Vicente D, Murakami S, Hui R, et al. Overall Survival with Durvalumab after Chemoradiotherapy in Stage III NSCLC. N Engl J Med. 2018 Dec 13;379(24):2342–50.

21. Hui R, Özgüroğlu M, Villegas A, Daniel D, Vicente D, Murakami S, et al. Patient-reported outcomes with durvalumab after chemoradiotherapy in stage III, unresectable non-small-cell lung cancer (PACIFIC): a randomised, controlled, phase 3 study. Lancet Oncol. 2019 Dec;20(12):1670–80.

22. Schadendorf D, Larkin J, Wolchok J, Hodi FS, Chiarion-Sileni V, Gonzalez R, et al. Health-related quality of life results from the phase III CheckMate 067 study. Eur J Cancer. 2017 Sep;82:80–91.

23. Larkin J, Minor D, D’Angelo S, Neyns B, Smylie M, Miller WHJ, et al. Overall Survival in Patients With Advanced Melanoma Who Received Nivolumab Versus Investigator’s Choice Chemotherapy in CheckMate 037: A Randomized, Controlled, Open-Label Phase III Trial. J Clin Oncol. 2018 Feb 1;36(4):383–90.

24. Robert C, Long GV, Brady B, Dutriaux C, Di Giacomo AM, Mortier L, et al. Five-Year Outcomes With Nivolumab in Patients With Wild-Type BRAF Advanced Melanoma. J Clin Oncol. 2020 Nov 20;38(33):3937–46.

25. Larkin J, Chiarion-Sileni V, Gonzalez R, Grob JJ, Rutkowski P, Lao CD, et al. Five-Year Survival with Combined Nivolumab and Ipilimumab in Advanced Melanoma. N Engl J Med. 2019 Oct 17;381(16):1535–46.

26. Ascierto PA, Del Vecchio M, Mandalá M, Gogas H, Arance AM, Dalle S, et al. Adjuvant nivolumab versus ipilimumab in resected stage IIIB-C and stage IV melanoma (CheckMate 238): 4-year results from a multicentre, double-blind, randomised, controlled, phase 3 trial. Lancet Oncol. 2020 Nov;21(11):1465–77.

27. Hellmann MD, Paz-Ares L, Bernabe Caro R, Zurawski B, Kim SW, Carcereny Costa E, et al. Nivolumab plus Ipilimumab in Advanced Non-Small-Cell Lung Cancer. N Engl J Med. 2019 Nov 21;381(21):2020–31.

28. Reck M, Schenker M, Lee KH, Provencio M, Nishio M, Lesniewski-Kmak K, et al. Nivolumab plus ipilimumab versus chemotherapy as first-line treatment in advanced non-small-cell lung cancer with high tumour mutational burden: patient-reported outcomes results from the randomised, open-label, phase III CheckMate 227 trial. Eur J Cancer. 2019 Jul;116:137–47.

29. Paz-Ares L, Ciuleanu TE, Cobo M, Schenker M, Zurawski B, Menezes J, et al. First-line nivolumab plus ipilimumab combined with two cycles of chemotherapy in patients with non-small-cell lung cancer (CheckMate 9LA): an international, randomised, open-label, phase 3 trial. Lancet Oncol. 2021 Jan 18;

30. Brahmer J, Reckamp KL, Baas P, Crinò L, Eberhardt WEE, Poddubskaya E, et al. Nivolumab versus Docetaxel in Advanced Squamous-Cell Non–Small-Cell Lung Cancer. N Engl J Med. 2015 Jul 9;373(2):123–35.

31. Borghaei H, Paz-Ares L, Horn L, Spigel DR, Steins M, Ready NE, et al. Nivolumab versus Docetaxel in Advanced Nonsquamous Non-Small-Cell Lung Cancer. N Engl J Med. 2015 Oct 22;373(17):1627–39.

32. Wu YL, Lu S, Cheng Y, Zhou C, Wang J, Mok T, et al. Nivolumab Versus Docetaxel in a Predominantly Chinese Patient Population With Previously Treated Advanced NSCLC: CheckMate 078 Randomized Phase III Clinical Trial. J Thorac Oncol. 2019 May;14(5):867–75.

33. Reck M, Brahmer J, Bennett B, Taylor F, Penrod JR, DeRosa M, et al. Evaluation of health-related quality of life and symptoms in patients with advanced non-squamous non-small cell lung cancer treated with nivolumab or docetaxel in CheckMate 057. Eur J Cancer. 2018 Oct;102:23–30.

34. Baas P, Scherpereel A, Nowak AK, Fujimoto N, Peters S, Tsao AS, et al. First-line nivolumab plus ipilimumab in unresectable malignant pleural mesothelioma (CheckMate 743): a multicentre, randomised, open-label, phase 3 trial. Lancet. 2021 Jan 30;397(10272):375–86.

35. Motzer RJ, Escudier B, George S, Hammers HJ, Srinivas S, Tykodi SS, et al. Nivolumab versus everolimus in patients with advanced renal cell carcinoma: Updated results with long-term follow-up of the randomized, open-label, phase 3 CheckMate 025 trial. Cancer. 2020 Sep 15;126(18):4156–67.

36. Cella D, Grünwald V, Nathan P, Doan J, Dastani H, Taylor F, et al. Quality of life in patients with advanced renal cell carcinoma given nivolumab versus everolimus in CheckMate 025: a randomised, open-label, phase 3 trial. Lancet Oncol. 2016 Jul;17(7):994–1003.

37. Motzer RJ, Tannir NM, McDermott DF, Arén Frontera O, Melichar B, Choueiri TK, et al. Nivolumab plus Ipilimumab versus Sunitinib in Advanced Renal-Cell Carcinoma. N Engl J Med. 2018 Apr 5;378(14):1277–90.

38. Cella D, Grünwald V, Escudier B, Hammers HJ, George S, Nathan P, et al. Patient-reported outcomes of patients with advanced renal cell carcinoma treated with nivolumab plus ipilimumab versus sunitinib (CheckMate 214): a randomised, phase 3 trial. Lancet Oncol. 2019 Feb;20(2):297–310.

39. Harrington KJ, Ferris RL, Blumenschein GJ, Colevas AD, Fayette J, Licitra L, et al. Nivolumab versus standard, single-agent therapy of investigator’s choice in recurrent or metastatic squamous cell carcinoma of the head and neck (CheckMate 141): health-related quality-of-life results from a randomised, phase 3 trial. Lancet Oncol. 2017 Aug;18(8):1104–15.

40. Yen CJ, Kiyota N, Hanai N, Takahashi S, Yokota T, Iwae S, et al. Two-year follow-up of a randomized phase III clinical trial of nivolumab vs. the investigator’s choice of therapy in the Asian population for recurrent or metastatic squamous cell carcinoma of the head and neck (CheckMate 141). Head Neck. 2020 Oct;42(10):2852–62.

41. Chen LT, Satoh T, Ryu MH, Chao Y, Kato K, Chung HC, et al. A phase 3 study of nivolumab in previously treated advanced gastric or gastroesophageal junction cancer (ATTRACTION-2): 2-year update data. Gastric Cancer. 2020 May;23(3):510–9.

42. Kang YK, Boku N, Satoh T, Ryu MH, Chao Y, Kato K, et al. Nivolumab in patients with advanced gastric or gastro-oesophageal junction cancer refractory to, or intolerant of, at least two previous chemotherapy regimens (ONO-4538-12, ATTRACTION-2): a randomised, double-blind, placebo-controlled, phase 3 trial. Lancet. 2017 Dec 2;390(10111):2461–71.

43. Gillison ML, Blumenschein G, Fayette J, Guigay J, Colevas AD, Licitra L, et al. Long-term Outcomes with Nivolumab as First-line Treatment in Recurrent or Metastatic Head and Neck Cancer: Subgroup Analysis of CheckMate 141. Oncologist. 2022 Mar 4;27(2):e194–8.

44. Paz-Ares L, Vicente D, Tafreshi A, Robinson A, Soto Parra H, Mazières J, et al. A Randomized, Placebo-Controlled Trial of Pembrolizumab Plus Chemotherapy in Patients With Metastatic Squamous NSCLC: Protocol-Specified Final Analysis of KEYNOTE-407. Journal of Thoracic Oncology. 2020 Oct;15(10):1657–69.

45. Eggermont AMM, Blank CU, Mandala M, Long GV, Atkinson VG, Dalle S, et al. Longer Follow-Up Confirms Recurrence-Free Survival Benefit of Adjuvant Pembrolizumab in High-Risk Stage III Melanoma: Updated Results From the EORTC 1325-MG/KEYNOTE-054 Trial. J Clin Oncol. 2020 Nov 20;38(33):3925–36.

46. Shitara K, Van Cutsem E, Bang YJ, Fuchs C, Wyrwicz L, Lee KW, et al. Efficacy and Safety of Pembrolizumab or Pembrolizumab Plus Chemotherapy vs Chemotherapy Alone for Patients With First-line, Advanced Gastric Cancer: The KEYNOTE-062 Phase 3 Randomized Clinical Trial. JAMA Oncol. 2020 Oct 1;6(10):1571–80.

47. Reck M, Rodríguez-Abreu D, Robinson AG, Hui R, Csőszi T, Fülöp A, et al. Pembrolizumab versus Chemotherapy for PD-L1-Positive Non-Small-Cell Lung Cancer. N Engl J Med. 2016 Nov 10;375(19):1823–33.

48. Brahmer JR, Rodríguez-Abreu D, Robinson AG, Hui R, Csőszi T, Fülöp A, et al. Health-related quality-of-life results for pembrolizumab versus chemotherapy in advanced, PD-L1-positive NSCLC (KEYNOTE-024): a multicentre, international, randomised, open-label phase 3 trial. Lancet Oncol. 2017 Dec;18(12):1600–9.

49. Fradet Y, Bellmunt J, Vaughn DJ, Lee JL, Fong L, Vogelzang NJ, et al. Randomized phase III KEYNOTE-045 trial of pembrolizumab versus paclitaxel, docetaxel, or vinflunine in recurrent advanced urothelial cancer: results of >2 years of follow-up. Ann Oncol. 2019 Jun 1;30(6):970–6.

50. Vaughn DJ, Bellmunt J, Fradet Y, Lee JL, Fong L, Vogelzang NJ, et al. Health-Related Quality-of-Life Analysis From KEYNOTE-045: A Phase III Study of Pembrolizumab Versus Chemotherapy for Previously Treated Advanced Urothelial Cancer. J Clin Oncol. 2018 Jun 1;36(16):1579–87.

51. Cloughesy TF, Mochizuki AY, Orpilla JR, Hugo W, Lee AH, Davidson TB, et al. Neoadjuvant anti-PD-1 immunotherapy promotes a survival benefit with intratumoral and systemic immune responses in recurrent glioblastoma. Nat Med. 2019 Mar;25(3):477–86.

52. Schachter J, Ribas A, Long GV, Arance A, Grob JJ, Mortier L, et al. Pembrolizumab versus ipilimumab for advanced melanoma: final overall survival results of a multicentre, randomised, open-label phase 3 study (KEYNOTE-006). Lancet. 2017 Oct 21;390(10105):1853–62.

53. Gandhi L, Rodríguez-Abreu D, Gadgeel S, Esteban E, Felip E, De Angelis F, et al. Pembrolizumab plus Chemotherapy in Metastatic Non-Small-Cell Lung Cancer. N Engl J Med. 2018 May 31;378(22):2078–92.

54. Garassino MC, Gadgeel S, Esteban E, Felip E, Speranza G, Domine M, et al. Patient-reported outcomes following pembrolizumab or placebo plus pemetrexed and platinum in patients with previously untreated, metastatic, non-squamous non-small-cell lung cancer (KEYNOTE-189): a multicentre, double-blind, randomised, placebo-controlled, phase 3 trial. Lancet Oncol. 2020 Mar;21(3):387–97.

55. Finn RS, Ryoo BY, Merle P, Kudo M, Bouattour M, Lim HY, et al. Pembrolizumab As Second-Line Therapy in Patients With Advanced Hepatocellular Carcinoma in KEYNOTE-240: A Randomized, Double-Blind, Phase III Trial. J Clin Oncol. 2020 Jan 20;38(3):193–202.

56. Ryoo BY, Merle P, Kulkarni AS, Cheng AL, Bouattour M, Lim HY, et al. Health-related quality-of-life impact of pembrolizumab versus best supportive care in previously systemically treated patients with advanced hepatocellular carcinoma: KEYNOTE-240. Cancer. 2021 Mar 15;127(6):865–74.

57. Schmid P, Cortes J, Pusztai L, McArthur H, Kümmel S, Bergh J, et al. Pembrolizumab for Early Triple-Negative Breast Cancer. N Engl J Med. 2020 Feb 27;382(9):810–21.

58. André T, Shiu KK, Kim TW, Jensen BV, Jensen LH, Punt C, et al. Pembrolizumab in Microsatellite-Instability-High Advanced Colorectal Cancer. N Engl J Med. 2020 Dec 3;383(23):2207–18.

59. Powles T, Plimack ER, Soulières D, Waddell T, Stus V, Gafanov R, et al. Pembrolizumab plus axitinib versus sunitinib monotherapy as first-line treatment of advanced renal cell carcinoma (KEYNOTE-426): extended follow-up from a randomised, open-label, phase 3 trial. Lancet Oncol. 2020 Dec;21(12):1563–73.

60. Cortes J, Cescon DW, Rugo HS, Nowecki Z, Im SA, Yusof MM, et al. Pembrolizumab plus chemotherapy versus placebo plus chemotherapy for previously untreated locally recurrent inoperable or metastatic triple-negative breast cancer (KEYNOTE-355): a randomised, placebo-controlled, double-blind, phase 3 clinical trial. Lancet. 2020 Dec 5;396(10265):1817–28.

61. Herbst RS, Baas P, Kim DW, Felip E, Pérez-Gracia JL, Han JY, et al. Pembrolizumab versus docetaxel for previously treated, PD-L1-positive, advanced non-small-cell lung cancer (KEYNOTE-010): a randomised controlled trial. Lancet. 2016 Apr 9;387(10027):1540–50.

62. Cohen EEW, Soulières D, Le Tourneau C, Dinis J, Licitra L, Ahn MJ, et al. Pembrolizumab versus methotrexate, docetaxel, or cetuximab for recurrent or metastatic head-and-neck squamous cell carcinoma (KEYNOTE-040): a randomised, open-label, phase 3 study. Lancet. 2019 Jan 12;393(10167):156–67.

63. Shitara K, Özgüroğlu M, Bang YJ, Di Bartolomeo M, Mandalà M, Ryu MH, et al. Pembrolizumab versus paclitaxel for previously treated, advanced gastric or gastro-oesophageal junction cancer (KEYNOTE-061): a randomised, open-label, controlled, phase 3 trial. Lancet. 2018 Jul 14;392(10142):123–33.

64. de Castro GJ, Kudaba I, Wu YL, Lopes G, Kowalski DM, Turna HZ, et al. Five-Year Outcomes With Pembrolizumab Versus Chemotherapy as First-Line Therapy in Patients With Non-Small-Cell Lung Cancer and Programmed Death Ligand-1 Tumor Proportion Score ≥ 1% in the KEYNOTE-042 Study. J Clin Oncol. 2022 Oct 28;JCO2102885.

65. Sezer A, Kilickap S, Gümüş M, Bondarenko I, Özgüroğlu M, Gogishvili M, et al. Cemiplimab monotherapy for first-line treatment of advanced non-small-cell lung cancer with PD-L1 of at least 50%: a multicentre, open-label, global, phase 3, randomised, controlled trial. The Lancet. 2021 Feb;397(10274):592–604.
